# Long COVID 12 months after discharge: persistent symptoms in patients hospitalised due to COVID-19 and patients hospitalised due to other causes—a multicentre cohort study

**DOI:** 10.1186/s12916-022-02292-6

**Published:** 2022-02-23

**Authors:** Mario Rivera-Izquierdo, Antonio Jesús Láinez-Ramos-Bossini, Inmaculada Guerrero-Fernández de Alba, Rocío Ortiz-González-Serna, Álvaro Serrano-Ortiz, Nicolás Francisco Fernández-Martínez, Rafael Ruiz-Montero, Jorge A. Cervilla

**Affiliations:** 1 Department of Preventive Medicine and Public Health, University of Granada, Avenida de la Investigación 11, 18016 Granada, Spain; 2 Service of Preventive Medicine and Public Health, Hospital Universitario San Cecilio, Granada, Spain; 3 Instituto Biosanitario de Granada (ibs. GRANADA), Granada, Spain; 4 Service of Radiology, Hospital Universitario Virgen de las Nieves, Granada, Spain; 5 Service of Preventive Medicine and Public Health, Hospital Universitario Reina Sofía, Córdoba, Spain; 6 Preventive Medicine and Public Health Research Group, Maimonides Institute for Research in Biomedicine of Cordoba (IMIBIC), Córdoba, Spain; 7 Department of Psychiatry, University of Granada, Granada, Spain

**Keywords:** Persistent COVID-19, Sequelae, Cohort study, Long term, Follow-up

## Abstract

**Background:**

Long-term-specific sequelae or persistent symptoms (SPS) after hospitalisation due to COVID-19 are not known. The aim of this study was to explore the presence of SPS 12 months after discharge in survivors hospitalised due to COVID-19 and compare it with survivors hospitalised due to other causes.

**Methods:**

Prospective cohort study, the Andalusian Cohort of Hospitalised patients for COVID-19 (ANCOHVID study), conducted in 4 hospitals and 29 primary care centres in Andalusia, Spain. The sample was composed of 906 adult patients; 453 patients hospitalised due to COVID-19 (exposed) and 453 hospitalised due to other causes (non-exposed) from March 1 to April 15, 2020, and discharged alive. The main outcomes were (1) the prevalence of SPS at 12 months after discharge and (2) the incidence of SPS after discharge. Outcome data at 12 months were compared between the exposed and non-exposed cohorts. Risk ratios were calculated, and bivariate analyses were performed.

**Results:**

A total of 163 (36.1%) and 160 (35.3%) patients of the exposed and non-exposed cohorts, respectively, showed at least one SPS at 12 months after discharge. The SPS with higher prevalence in the subgroup of patients hospitalised due to COVID-19 12 months after discharge were persistent pharyngeal symptoms (*p*<0.001), neurological SPS (*p*=0.049), confusion or memory loss (*p*=0.043), thrombotic events (*p*=0.025) and anxiety (*p*=0.046). The incidence of SPS was higher for the exposed cohort regarding pharyngeal symptoms (risk ratio, 8.00; 95% CI, 1.85 to 36.12), confusion or memory loss (risk ratio, 3.50; 95% CI, 1.16 to 10.55) and anxiety symptoms (risk ratio, 2.36; 95% CI, 1.28 to 4.34).

**Conclusions:**

There was a similar frequency of long-term SPS after discharge at 12 months, regardless of the cause of admission (COVID-19 or other causes). Nevertheless, some symptoms that were found to be more associated with COVID-19, such as memory loss or anxiety, merit further investigation. These results should guide future follow-up of COVID-19 patients after hospital discharge.

**Supplementary Information:**

The online version contains supplementary material available at 10.1186/s12916-022-02292-6.

## Background

To date, more than 190 million cases of COVID-19 have been reported worldwide. Many sequelae or persistent symptoms (SPS) have been observed after hospitalisation, including persistent dyspnoea, fatigue or cognitive impairment [1–7]. Long-COVID has been defined as the persistence of symptoms 4 weeks after the acute infection [8], and it is of major current interest to the scientific community to improve follow-up and mitigate the side effects of the pandemic. Nevertheless, very few studies have reported long-term SPS 12 months after hospital discharge [9, 10], although the presence of SPS exposes healthcare systems and patients to a wide range of chronic symptoms and diseases [11]. In addition, most of the published studies have reported the prevalence of SPS, which could entail significant biases as many symptoms might have been present prior to COVID-19 infection or hospitalisation. Therefore, it is important to collect incident symptoms associated with the disease to avoid ascribing SPS to an equivocal cause.

Post-discharge syndrome has been defined as the persistence of symptoms in the most severe cases (i.e., those requiring hospitalisation) of COVID-19 after hospital discharge [12, 13]. These SPS might be caused by different factors such as a more severe course of the disease, hospital stay or treatments. Nevertheless, studies published to date, both regarding Long-COVID or post-discharge syndrome, have focused on the description and analysis of these cases, but no comparison (control) group has been used to determine the specific side effects of COVID-19 hospitalisation in comparison with other conditions. It is not possible to quantify which SPS are specific to COVID-19 and which are common in the general population (or, in this case, in patients requiring hospitalisation due to any cause) without a comparison group. For example, a recently published meta-analysis on long-COVID in children highlighted the critical importance of including a control group in studies on this topic, as the frequency of SPS tended to be similar when comparing COVID-19 with non-COVID-19 patients [14]. Therefore, to design cohort studies that add relevant evidence to the current literature, it is necessary to include a non-exposed cohort of patients without COVID-19.

The aim of this study was twofold. First, to describe the prevalence of SPS 12 months after hospitalisation for COVID-19, and its distribution by sex and age. Second, to compare the prevalence and incidence of SPS after discharge in patients hospitalised due to COVID-19 and in those hospitalised for other causes, to elucidate which symptoms are frequent after hospitalisation, and which are specific to COVID-19.

## Methods

### Study design and setting

We conducted a prospective, multicentre cohort study. The exposed cohort (patients hospitalised due to COVID-19) was selected from the Andalusian Cohort of Hospitalised patients for COVID-19 (ANCOHVID Study) [15]: a randomly selected sample from all hospitalised patients, admitted from March 1 to April 15, 2020, with laboratory-confirmed SARS-CoV-2 infection through nasopharyngeal polymerase chain reaction (PCR)-positive samples was included according to the optimal required sample size. This sample consisted of patients admitted to four hospitals in Andalusia, Spain (Córdoba, Jaén, Granada and Puerto Real). The non-exposed cohort (patients hospitalised due to other causes) were matched by institution and date of admission. We decided not to match for other factors since our aim was to compare COVID-19 patients with a representative sample of those hospitalised due to other causes, regardless of their differences in baseline characteristics. Institution and date of admission were considered to select the comparison group from the same cohort of patients of the exposure group (patients from the same area at the same time).

The sample size for cohort studies was calculated using the Poisson approximation. For a two-tailed test with an alpha error of 0.05 and a beta error of 0.2, it was estimated that a total of 453 exposed and 453 non-exposed (*n* = 906) were needed to detect a minimum risk ratio of 1.2, assuming a 50% prevalence of SPS at 12 months according to the only study published to date with this follow-up duration [10]. We assumed a 10% drop-out rate during the 12-month follow-up. Patients discharged alive in both groups were included and followed up 12 months after discharge. Patients lost to follow-up were excluded from the analyses. The study followed the recommendations of the Strengthening the Reporting of Observational Studies in Epidemiology (STROBE) guidelines (Additional file 1).

### Data collection and variables

Data were collected from the Spanish modified version of the open-access Case Report Form of the Clinical Characterization Protocol for Severe Emerging Infections of the International Severe Acute Respiratory and Emerging Infection Consortium (ISARIC). For the exposed cohort, adult patients (≥ 18 years) were identified by daily listing of positive PCR results from nasopharyngeal swabs reported by the microbiology services of the participating hospitals. In addition, to select the non-exposed cohort, a professional search was requested from the Clinical Documentation services of the centres and the Epidemiological Surveillance System of Andalusia (SVEA). Exposure was considered as hospitalisation due to confirmed COVID-19. The outcomes were SPS at 12 months and incident SPS after hospital discharge. Data on the sociodemographic and clinical characteristics of patients were consulted in the medical records. To collect information on SPS after discharge, patients were consulted by telephone 12 months after discharge. Patients were asked about (1) the prevalence of SPS at 12 months and (2) incidence of new SPS after discharge. All patients were questioned by the same trained interviewer, who was blinded to patient exposure. Patients were informed that the aims of the study were to collect SPS after hospitalisation for any cause, but no emphasis was placed on COVID-19 to avoid potential biases. The consideration of each SPS to distinguish between prevalent, incident and previous symptoms is specified in Additional file 2: Fig. S1. These data were supplemented by consulting reports scheduled from primary care after discharge, all of which were standardised and registered in the clinical histories, covering a total of 29 primary care centres in our sample. Regarding the incidence of SPS, symptoms that were present prior to hospital admission for COVID-19 were excluded. The variables included in the study were sex, age, institution, days of hospitalisation, admission to the intensive care unit, medical history and SPS. Age was considered quantitatively, except for graphical analyses, where intervals of age according to previous studies [15] were grouped. The SPS collected were divided in subgroups according to previous studies [15] as follows: general or systemic SPS (including fatigue, muscle weakness and muscle or joint pain), respiratory SPS (including dyspnoea, chest pain and persistent pharyngeal symptoms which included sore throat, persistent cough or dysphonia), neurological SPS (headache, sensitivity disorders, movement disorders, and confusion or memory loss), mental health SPS (including depressive symptoms, anxiety symptoms and sleep disturbances), haematological SPS (including thrombotic events), dermatological SPS, nephrological SPS, urological SPS, otorhinolaryngological SPS, ophthalmological SPS and digestive SPS (including diarrhoea, constipation and abdominal pain).

### Statistical analysis

Continuous variables were described using means (standard deviation) or medians (interquartile range), as appropriate. Categorical variables were described using count (percentage). Comparisons of the prevalence of SPS 12 months after hospital discharge between the exposed and non-exposed cohorts were performed using chi-square or Fisher’s exact tests, as appropriate. The distribution of SPS by sex and age in the exposed cohort was graphically analysed using Sankey diagrams and heat maps, respectively. The diagrams were obtained using the *ggplot* and *ggalluvial* packages from R software. Detailed information on the commands used for the diagrams is available as Additional file 2: Table S1. Then, logistic regression models for each SPS prevalence were applied using sex, age, comorbidities and ICU admission as covariates. Incidences of each SPS in both groups were subsequently quantified. Risk ratios (cumulative incidence of each SPS in the exposed cohort/cumulative incidence of the same SPS in the non-exposed cohort) and their corresponding 95% confidence intervals (CI) were calculated. All statistical analyses were performed using R version 4.0.2 (R Core Team (2020). R: A language and environment for statistical computing. R Foundation for Statistical Computing, Vienna, Austria. URL https://www.R-project.org).

### Ethical considerations

The protocol was approved by the Institutional Review Board of the Provincial Research Ethics Committee of Granada on October 1, 2020, and was endorsed by the review boards of the participating centres (Provincial Research Ethical Committees of Cádiz, Córdoba and Jaén). The ethical implications of the study were considered according to the principles of the Declaration of Helsinki. The database was anonymised, and no identification data were used in the analyses.

## Results

**Fig. 1:**
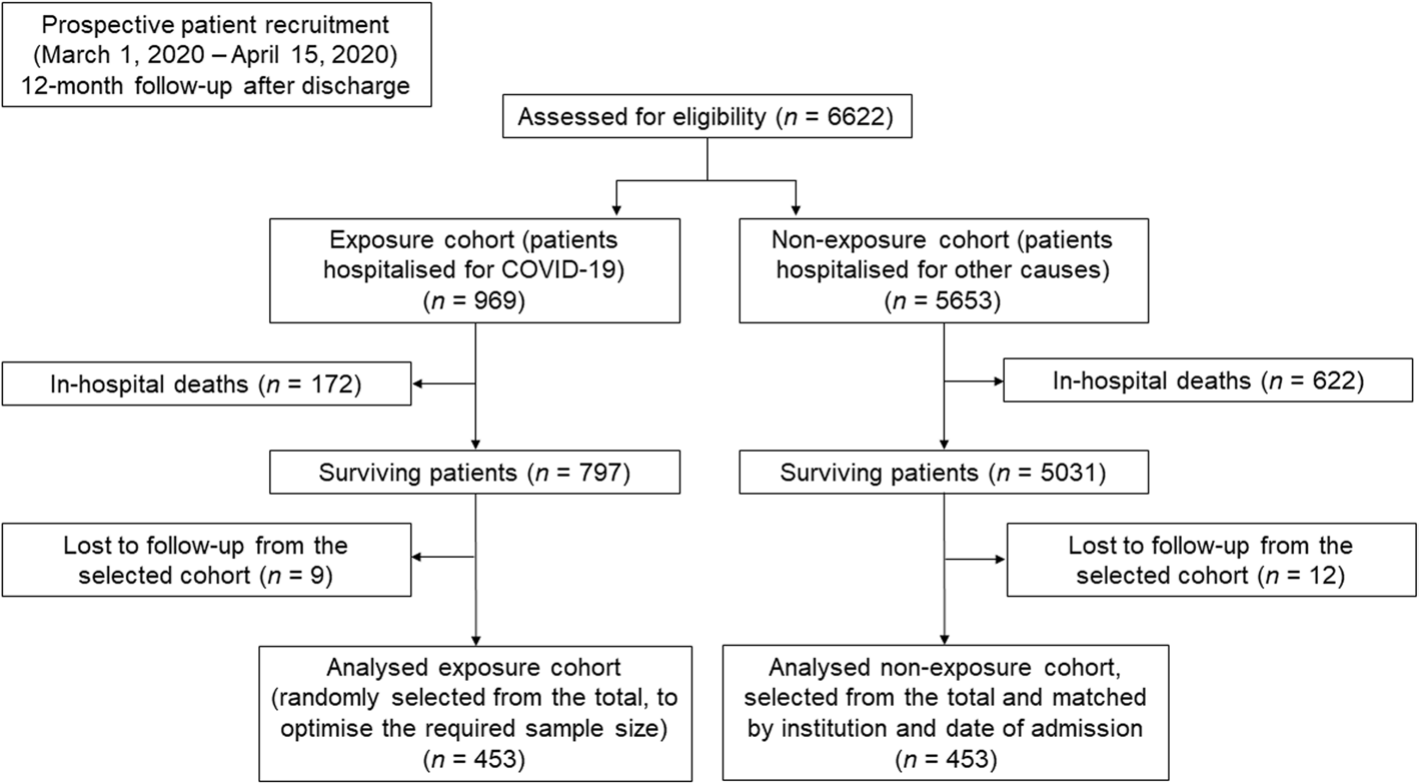
Flow chart of patient selection for the cohort study according to STROBE guidelines

Figure 1 shows the flow chart for sample selection (*n* = 906). There was a total of 5653 patients hospitalised due to causes other than COVID-19 in the study period. Of these, 453 patients were randomly selected and matched to the exposed cohort by institution and date of admission. The causes of admission in the non-exposed cohort are summarised in Additional file 2: Table S2. Of the 453 patients hospitalised due to COVID-19, 163 (36.1%) reported at least one SPS at 12 months after discharge, compared with 160 (35.3%) of the 453 patients hospitalised due to other causes. The main characteristics of the sample, stratified by cause of admission, are presented in Table 1. COVID-19 patients showed a higher proportion of men and older age, and similar number of days of hospitalisation and intensive care admission, compared to patients hospitalised due to other causes. No sociodemographic factors (sex, age and centre) were associated with the development of SPS, neither in the total sample nor in the exposed cohort.

**Table 1:** Sociodemographic and in-hospital characteristics of the sample stratified by cause of admission (COVID-19 vs other causes)

Characteristic	Exposed cohort (hospitalised due to COVID-19) (***n*** = 453)	Non-exposed cohort (hospitalised due to other causes) (***n*** = 453)	***P***-value^1^
	***n*** **(%)/*x*** **(s)**	***n*** **(%)/*x*** **(s)**	
Age, *x* (s)	61.2 (14.3)	55.9 (17.8)	<0.001
Sex, *n* (%)			
Men	260 (57.4)	211 (46.6)	0.001
Women	193 (42.6)	242 (53.2)	
Comorbidities, *n* (%)	306 (67.5)	297 (65.6)	0.872
Dependency in activities of daily living (patients requiring help), *n* (%)	68 (15.0)	59 (13.0)	0.188
Centre, *n* (%)			
HUCSC, Granada	161 (35.5)	161 (35.5)	-
HURS, Córdoba	125 (27.6)	125 (27.6)	
HUPR, Cádiz	19 (4.2)	19 (4.2)	
CHJ, Jaén	148 (32.7)	148 (32.7)	
Days of hospitalisation, *x* (s)	15 (13.5)	13 (11.6)	0.451
Intensive care admission, *n* (%)	48 (10.6)	41 (9.1)	0.081

^1^*P*-value of T test for continuous variables and chi-square test for categorical variables

### Prevalence of SPS 12 months after hospital discharge

**Fig. 2:**
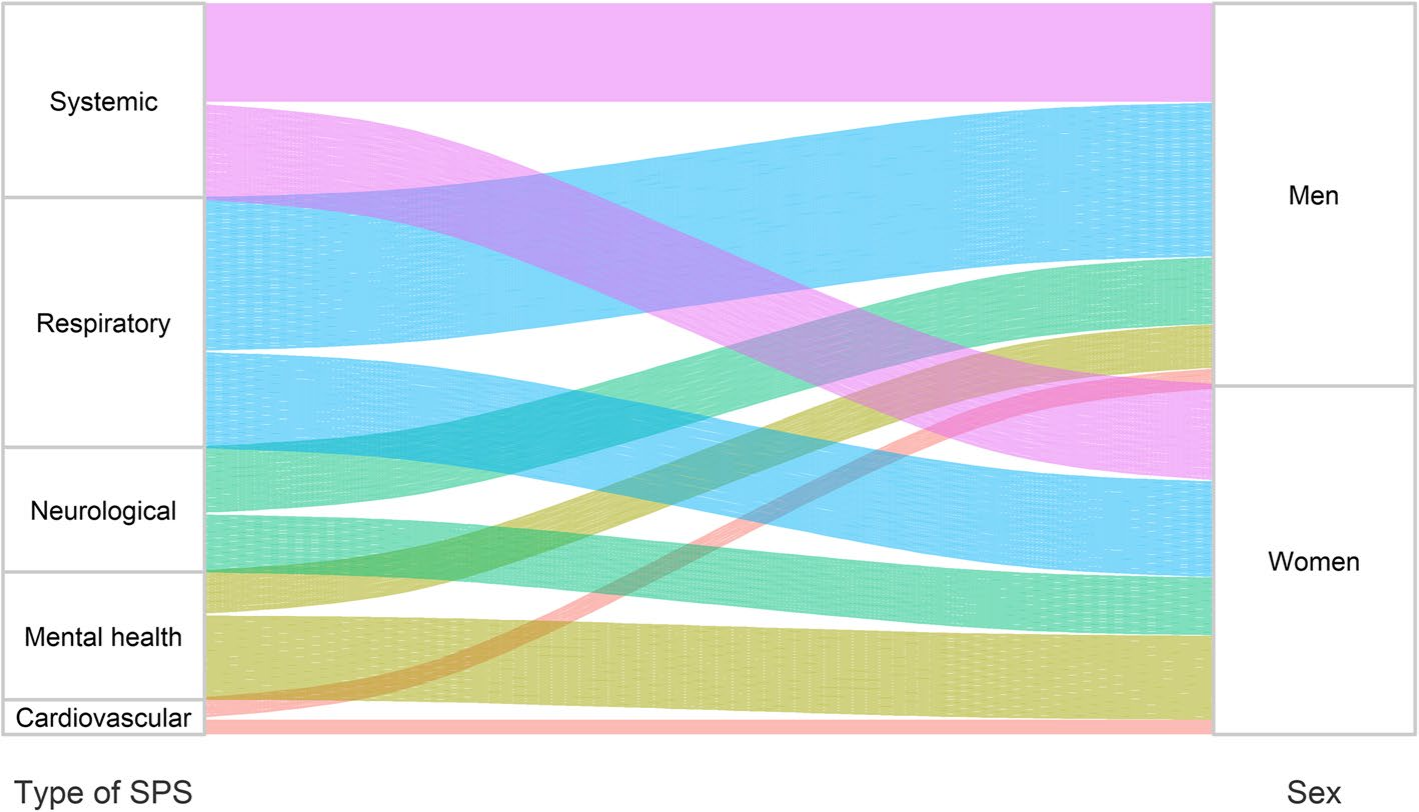
Sankey diagram of the prevalence of sequelae and persistent symptoms (SPS) 12 months after discharge from hospitalisation due to COVID-19, stratified by sex. The five most frequent SPS are represented in different colours. The size (width) of each line presented in the diagram is proportional to the quantity of patients who showed this SPS distributed by sex

**Fig. 3:**
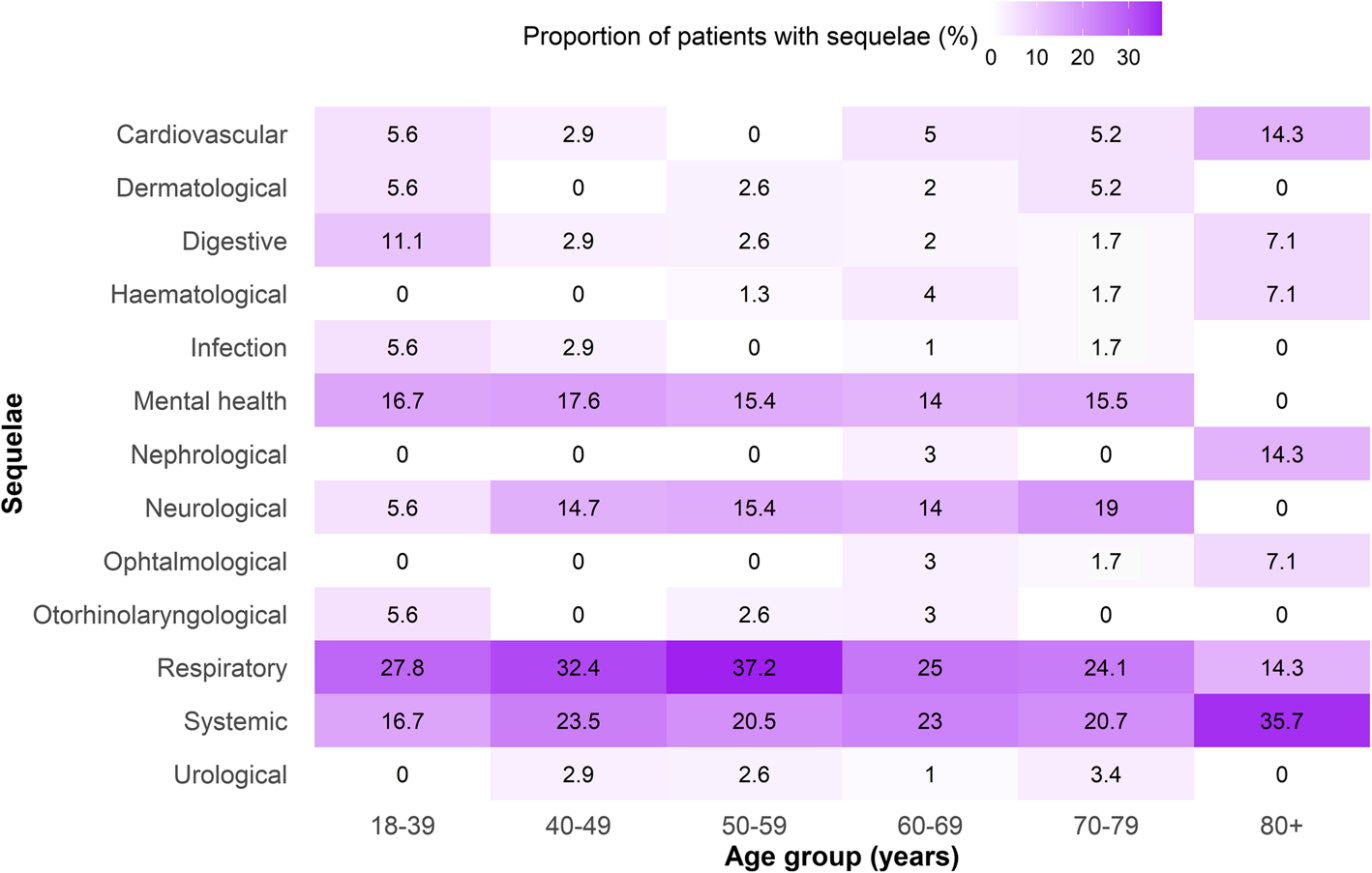
Heat map of the prevalence of sequelae and persistent symptoms (SPS) 12 months after discharge from hospitalisation due COVID-19, stratified by age groups. SPS are grouped by systems in the rows. Age groups are presented in the columns. The relative proportion of patients that reported each SPS is shown within the cells. Darker colour indicates a higher proportion of SPS at 12 months after discharge

The prevalence of the most frequent long-term SPS reported 12 months after hospital discharge in the exposed cohort is illustrated in Figs. 2 and 3. The distribution by sex is presented in a Sankey diagram (Fig. 2). This figure shows the distribution of the five most frequent types of SPS (cardiovascular, mental health, neurological, respiratory and systemic). The larger size (width) of each line represents a higher proportion of patients presenting each SPS. As shown in the figure, a higher frequency of respiratory and systemic SPS was observed in men, and a higher prevalence of mental health SPS (especially anxiety and depressive symptoms) was found in women. The distribution of SPS at 12 months by age group is presented graphically in a heat map (Fig. 3). This figure shows the relative contribution of each type of SPS to the total number of SPS, per age group: the colour intensity is proportional to the weight of each type of SPS, therefore adding up to 100% in every age group. The graph shows that older patients exhibited a higher prevalence of cardiovascular, neurological and systemic SPS, whilst younger patients showed a higher frequency of mental health and digestive SPS. The associations between different SPS are shown in a correlation matrix (Additional file 2: Fig. S2). Only anxiety and depression (*R*=0.49), and dyspnoea and pharyngeal symptoms (*R*=0.33) showed relevant correlations.

The frequency of specific SPS in the exposed and non-exposed cohorts is represented in Table 2. The most frequent SPS at 12 months in COVID-19 patients were respiratory (19.2%)—especially dyspnoea (15.5%)—, general or systemic SPS (15.0%)—especially fatigue (8.2%)—, neurological SPS (9.7%) and mental health SPS (10.6%) —especially anxiety (7.3%)—. Briefly, the most frequently occurring SPS in the exposed cohort (COVID-19) were persistent pharyngeal symptoms (*p*<0.001), general neurological SPS (*p*=0.049), confusion or memory loss (*p*=0.043), thrombotic events (*p*=0.025) and anxiety (*p*=0.046). The SPS most frequently associated with non-COVID-19 conditions were fatigue (*p*=0.038), dermatological SPS (*p*=0.008), urological SPS (*p*=0.031), ophthalmological SPS (*p*=0.015) and digestive SPS (*p*<0.001), especially diarrhoea (*p*=0.007) and abdominal pain (*p*=0.007). The results of adjusted logistic regression models can be found in Table 3. Briefly, the exposed and non-exposed cohorts showed no overall differences regarding the presence of SPS. Nevertheless, patients hospitalised due to COVID-19 showed a higher prevalence of respiratory SPS (OR, 1.47; 95% CI, 1.00 to 2.46), neurological SPS (OR, 2.20; 95% CI, 1.21 to 3.96) and anxiety symptoms (OR, 1.56; 95% CI, 1.08 to 2.04), after adjusting for sex, age, ICU admission and baseline comorbidities.

**Table 2:** Prevalence of sequelae or persistent symptoms (SPS) 12 months after discharge

SPS	Exposed cohort (hospitalised due to COVID-19) (***n*** = 453)	Non-exposed cohort (hospitalised due to other causes) (***n*** = 453)	***P***-value^1^
	***n*** (%)	***n*** (%)	
Any SPS	163 (36.1)	160 (35.3)	0.797
General/systemic SPS	68 (15.0)	80 (17.7)	0.281
Fatigue	37 (8.2)	56 (12.4)	0.038
Muscle weakness	14 (3.1)	8 (1.8)	0.195
Muscle or joint pain	42 (9.3)	48 (10.6)	0.505
Respiratory SPS	87 (19.2)	72 (15.9)	0.190
Dyspnoea	70 (15.5)	56 (12.4)	0.179
Chest pain	5 (1.1)	8 (1.8)	0.578
Pharyngeal symptoms	16 (3.5)	2 (0.4)	<0.001
Neurological SPS	44 (9.7)	28 (6.2)	0.049
Headache	13 (2.9)	12 (2.6)	0.839
Sensitivity disorders	9 (2.0)	8 (1.8)	0.807
Movement disorders	5 (1.1)	1 (0.2)	0.062
Confusion, memory loss	16 (3.5)	8 (1.8)	0.043
Mental health SPS	48 (10.6)	46 (10.2)	0.828
Depressive symptoms	22 (4.9)	20 (4.4)	0.752
Anxiety symptoms	33 (7.3)	19 (4.2)	0.046
Sleep disturbances	17 (3.8)	14 (3.1)	0.584
Haematological SPS	7 (1.5)	7 (1.5)	1.000
Thrombotic events	5 (1.1)	0 (0.0)	0.025
Dermatological SPS	9 (2.0)	24 (5.3)	0.008
Nephrological SPS	5 (1.1)	2 (0.4)	0.162
Urological SPS	6 (1.3)	16 (3.5)	0.031
Otorhinolaryngological SPS	6 (1.3)	8 (1.8)	0.590
Ophthalmological SPS	5 (1.1)	16 (3.5)	0.015
Digestive SPS	9 (2.0)	32 (7.1)	<0.001
Diarrhoea	4 (0.9)	16 (3.5)	0.007
Constipation	3 (0.7)	8 (1.8)	0.129
Abdominal pain	4 (0.9)	16 (3.5)	0.007
Infection	7 (1.5)	6 (1.3)	0.898

^1^*P*-value of chi-squared test (if conditions of application were met) or Fisher’s exact test, as appropriate

**Table 3:** Logistic regression models comparing the prevalence of sequelae and persistent symptoms (SPS) in patients hospitalised due to COVID-19 compared with patients hospitalised due to other causes (reference group). Multivariate models included sex, age, ICU admission and comorbidities as covariates

SPS^1^	OR	95%CI
Any SPS	1.13	0.85 to 1.51
General/systemic SPS	0.82	0.56 to 1.19
Fatigue	0.57	0.36 to 0.90
Muscle or joint pain	0.82	0.52 to 1.30
Respiratory SPS	1.47	1.00 to 2.16
Dyspnoea	1.13	0.76 to 1.68
Neurological SPS	2.20	1.21 to 3.96
Headache	1.28	0.52 to 3.13
Sensitivity disorders	0.82	0.31 to 2.17
Confusion, memory loss	1.83	0.74 to 4.81
Mental health SPS	1.21	0.75 to 2.27
Depressive symptoms	1.08	0.89 to1.28
Anxiety symptoms	1.56	1.08 to 2.04
Sleep disturbances	1.11	0.88 to 1.48
Dermatological SPS	0.44	0.19 to 1.00
Digestive SPS	0.28	0.13 to 0.62

^1^Only SPS with enough number of patients (>8 per group) were included in this analysis

### Incidence of SPS from hospital discharge to 12 months after discharge

Table 4 shows the incidence of SPS after discharge in the exposed and non-exposed cohorts. In general, the incidence of each SPS was lower than the prevalence, given that several of the SPS collected at 12 months after discharge were present before the cause of hospitalisation, except for digestive SPS (high incidence in COVID-19 patients but low prevalence at 12 months). The incidences most associated with COVID-19 hospitalisation were persistent pharyngeal symptoms (RR, 8.00; 95% CI, 1.85 to 36.12), confusion or memory loss (RR, 3.50; 95% CI, 1.16 to 10.55), and anxiety symptoms (RR, 2.36; 95% CI, 1.28 to 4.34).

**Table 4:** Incidences of sequelae or persistent symptoms (SPS) after discharge

SPS	Exposed cohort (hospitalised due to COVID-19) (***n*** = 453)	Non-exposed cohort (hospitalised due to other causes) (***n*** = 453)	Risk ratio (95% CI)^**b**^
	***N*** (Cumulative incidence)^**a**^	***N*** (Cumulative incidence)^**a**^	
Any SPS	120 (26.5)	105 (23.2)	1.14 (0.91 to 1.43)
General/systemic SPS	55 (12.1)	63 (13.9)	0.87 (0.62 to 1.22)
Fatigue	35 (7.7)	40 (8.8)	0.88 (0.57 to 1.35)
Muscle weakness	14 (3.1)	8 (1.8)	1.75 (0.74 to 4.27)
Muscle or joint pain	35 (7.7)	30 (6.6)	1.17 (0.73 to 1.87)
Respiratory SPS	77 (17.0)	58 (12.8)	1.33 (0.97 to 1.82)
Dyspnoea	60 (13.2)	43 (9.5)	1.40 (0.96 to 2.02)
Chest pain	4 (0.9)	6 (1.3)	0.67 (0.19 to 2.37)
Pharyngeal symptoms	16 (3.5)	2 (0.4)	8.00 (1.85 to 36.12)
Neurological SPS	40 (8.8)	25 (5.5)	1.60 (0.99 to 2.59)
Headache	9 (2.0)	9 (2.0)	1.00 (0.40 to 2.50)
Sensitivity disorders	8 (1.8)	7 (1.5)	1.14 (0.42 to 3.12)
Movement disorders	3 (0.7)	1 (0.2)	3.00 (0.30 to 28.70)
Confusion, memory loss	14 (3.1)	4 (0.9)	3.50 (1.16 to 10.55)
Mental health SPS	41 (9.1)	35 (7.7)	1.17 (0.76 to 1.80)
Depressive symptoms	18 (4.0)	16 (3.5)	1.13 (0.58 to 2.18)
Anxiety symptoms	33 (7.3)	14 (3.1)	2.36 (1.28 to 4.34)
Sleep disturbances	10 (2.2)	7 (1.5)	1.43 (0.55 to 3.81)
Haematological SPS	5 (1.1)	4 (0.9)	1.25 (0.34 to 4.62)
Dermatological SPS	8 (1.8)	18 (4.0)	0.46 (0.21 to 1.02)
Nephrological SPS	5 (1.1)	2 (0.4)	2.20 (0.50 to 9.76)
Urological SPS	5 (1.1)	12 (2.6)	0.44 (0.16 to 1.19)
Otorhinolaryngological SPS	4 (0.9)	6 (1.3)	0.69 (0.21 to 2.29)
Ophthalmological SPS	4 (0.9)	14 (3.1)	0.31 (0.11 to 0.89)
Digestive SPS	42 (9.3)	45 (9.9)	0.30 (0.14 to 0.63)
Diarrhoea	35 (7.7)	33 (7.3)	0.93 (0.63 to 1.39)
Constipation	6 (1.3)	9 (2.0)	0.68 (0.25 to 184)
Abdominal pain	12 (2.6)	21 (4.6)	0.58 (0.29 to 1.15)
Infection	7 (1.5)	6 (1.3)	1.15 (0.41 to 3.27)

^a^Cumulative incidence was calculated as new cases of SPS (not present before hospitalisation)/all susceptible patients

^b^The non-exposed cohort was used as reference group for risk ratios. Risk ratios show the differences in cumulative incidences (unadjusted) between the exposed and non-exposed cohorts

## Discussion

We presented a 12-month follow-up multicentre, prospective cohort study comparing patients discharged alive from hospitalisation due to COVID-19 and patients discharged alive due to other causes. We collected detailed information on the prevalence of SPS at 12 months after hospital discharge and the incidence of SPS not present before the cause of hospitalisation. Our findings indicate that patients who required hospitalisation showed a similar frequency of SPS, regardless of the cause (i.e., COVID-19 or other). Thus, approximately one third of patients reported persistent symptoms at 12 months, and one quarter of patients reported incident SPS not present prior to hospitalisation. These findings suggest that, rather than attributing persistent symptoms to COVID-19, it is the need for hospitalisation that prolongs long-term symptomatology (SPS after discharge). Nevertheless, our data showed that the incidences of three persistent symptoms are specifically associated with COVID-19 (Long-COVID): persistent pharyngeal symptoms, confusion or memory loss, and anxiety symptoms. Also, the results of the multivariate models adjusted for sociodemographic and clinical characteristics showed that the prevalence of respiratory SPS, neurological SPS and anxiety was higher in patients hospitalised due to COVID-19.

In our study, dyspnoea, fatigue and anxiety were the most frequent SPS 12 months after discharge. Specifically, 13.2% of patients presented incident dyspnoea and 15.5% showed dyspnoea at 12 months. These data agree with other authors who reported a prevalence of dyspnoea at 1 year after hospital discharge of 15% [9], but contrast with other studies reporting widely variable prevalence values ranging from 4.6% at 5 weeks [2] to 37.5% at 12 months [10]. We observed that 12.4% of patients requiring hospitalisation due to other causes had dyspnoea. Regarding fatigue, our findings showed 7.7% of incident cases and a prevalence of 8.2% at 12 months. These figures are lower than those reported in other series [9, 10]. Accordingly, our data indicate that dyspnoea and fatigue are common causes of SPS after hospitalisation but are not specific to COVID-19. Regarding mental health SPS, we found that patients hospitalised due to COVID-19 had a higher prevalence and incidence of anxiety than patients hospitalised for other causes. Importantly, this anxiety might be a biological sequela of COVID-19 or, more likely, a consequence of the health and social situation during the pandemic, especially because of the restrictions that were applied during hospitalisation in the exposed cohort [16]. In our opinion, persistent anxiety could influence the degree of self-observation and the importance of the reported SPS and could even increase the presence of somatic symptoms, as previously reported [17]. Therefore, our results suggest that follow-up of anxiety SPS in patients discharged alive from hospitalisation due to COVID-19 should be strengthened to mitigate the long-term side-effects of the pandemic. Another relevant aspect that should be explored in future research is the association between hospital length or invasive treatments with SPS (especially anxiety) after discharge. Although we have not explored this association in this study, we believe that the identification of predictors of anxiety could help to design effective preventive measures.

We also found an interesting association with confusion or memory loss in the exposed cohort, which was particularly higher in older patients. As reported by other authors, concentration difficulties [10] and cognitive impairment [2] have been widely associated with COVID-19. In all, some degree of post-COVID-19 specificity for our findings in both mental health and neurological SPS seems plausible as our results concord to previous reports [18]. Our findings support these observations, as COVID-19 patients showed higher rates of memory loss than those hospitalised due to other causes (including patients from Neurology or Mental Health services). If the scientific literature confirms this association, it could be important to provide additional community services at discharge to help patients with this SPS. For example, multidisciplinary teams composed of occupational therapists, expert clinicians (e.g., neurologists or primary care physicians), community rehabilitators or social workers might help these patients recover more quickly and effectively from this COVID-19 sequela.

Finally, the persistence of pharyngeal symptoms 12 months after discharge was higher in COVID-19 patients.

The required sample size was calculated to optimise resources. In addition, we attempted to overcome the shortcomings of the studies published to date. Accordingly, we collected data on long-term SPS at 12 months after hospital discharge. However, to avoid biassing the results, our focus was not limited to collecting the prevalence of SPS, but also the incidence of new symptoms, as many SPS that have been attributed to COVID-19 may also be prevalent in the general population regardless of the type of disease. A comparison group (patients hospitalised due to other causes) was also used. Ideally, COVID-19 patients of the general population should be compared with people from the community with no history of COVID-19 in a population-based cohort study. Nevertheless, due to feasibility criteria and given that we only had access to hospitalised patients in our registries, we decided to design a hospital-based cohort study, comparing COVID-19 patients with patients hospitalised due to other causes and randomly selected, matched by institution and date of admission. No other factors were used to match the non-exposed cohort because our aim was to obtain a representative sample of all patients hospitalised due to causes other than COVID-19. As baseline characteristics were different between the exposed and non-exposed cohort and, in order to adjust for these potential confounders, we estimated multivariate logistic regression models for the prevalence of SPS. Regarding incidences, unadjusted risk ratios were presented, but they should be interpreted with caution. We studied a large cohort from Spain, but these data should be supplemented with data from other countries and health services to increase its generalisability. Although we achieved the a priori calculated sample size for our cohort study, several significantly higher risk ratios (Table 4) occurred in a very low number of patients; therefore, these results should be considered cautiously. A perceived limitation of our study is that we collected the reported SPS by telephone. Some patients (especially those older or cognitively impaired) were unable to answer and, therefore, a family member reported the SPS. Although these data might not be complete, we supplemented them with information collected in routine primary care follow-up consultations. The exposed and non-exposed cohorts were not completely alike in terms of their baseline characteristics (age and sex). However, we do not consider this to be a major limitation, as the aim of the study was to compare patients hospitalised due to COVID-19 with those hospitalised due to other causes, and these populations would not necessarily match in terms of baseline characteristics. Finally, we only collected the number of comorbidities in the comparison groups; however, the analysis of differences in specific baseline comorbidities and their role on the development of SPS might be of interest for future research.

Our results should guide future follow-up strategies on long-COVID and post-discharge COVID-19 patients. Future studies conducted 12 months after hospitalisation should explore the prevalence and incidence of COVID-19 SPS in other countries. Moreover, studies conducted in population-based cohorts should compare COVID-19 and non-COVID-19 patients to corroborate the results observed in hospitalised patients. Knowledge of symptoms specific to COVID-19 after hospital discharge should serve to enhance quality information to patients, early detection of symptoms, and further research on effective treatments. Of note, clinicians and researchers should consider the relevance of anxiety and memory loss in patients previously hospitalised due to COVID-19 to mitigate the long-term effects of the pandemic on population health.

## Conclusions

In this cohort of patients discharged after hospitalisation due to COVID-19 and due to other causes, with a 12-month follow-up, prevalent and incident SPS were varied and frequent regardless of the cause of admission. There were no significant differences regarding the presence of most SPS at 12 months after hospital discharge between the two subgroups. However, the distribution of SPS by sex and age groups varied, and COVID-19 patients showed higher rates of persistent pharyngeal symptoms, confusion or memory loss, and anxiety symptoms. These data should be further supplemented by population-based cohort studies.

## Supplementary Information

**Additional file 1.** STROBE checklist. Checklist of items included in reports of observational studies.

**Additional file 2: Table S1.** Causes of admission of the non-exposed cohort (*n* = 453). **Table S2.** Commands used for the diagrams with R software. **Fig. S1.** Consideration of each reported sequelae or persistent symptoms (SPS) collected by telephone to distinguish between previous, prevalent, and incident SPS. **Fig. S2.** Correlation matrix of the association between the most frequent sequelae and persistent symptoms (SPS).

## Data Availability

The data and materials supporting the conclusions of this article are available from the corresponding author on reasonable request.

## Abbreviations

SPS: Sequelae or persistent symptoms
ANCOHVID: Andalusian Cohort of Hospitalised Patients for COVID-19
PCR: Polymerase chain reaction
STROBE: Strengthening the Reporting of Observational Studies in Epidemiology
ISARIC: International Severe Acute Respiratory and Emerging Infection Consortium
SVEA: Epidemiologic Surveillance System of Andalusia
CI: Confidence interval
