# Supplementary material for: Long COVID 12 months after discharge: persistent symptoms in patients hospitalised due to COVID-19 and patients hospitalised due to other causes—a multicentre cohort study
Source: BMC Med. 2022 Feb 23;20:92. doi: 10.1186/s12916-022-02292-6 (PMC8863509; doi:10.1186/s12916-022-02292-6)
Supplement: Supplementary file 1 — Additional file 1. STROBE checklist. Checklist of items included in reports of observational studies. [file 12916_2022_2292_MOESM1_ESM.doc]

**Additional File 1.** STROBE checklist. Checklist of items included in reports of observational studies

|  | Item No | Recommendation |
| --- | --- | --- |
| **Title and abstract** | 1 **Pages 1 and 3-4 abstract** | (*a*) Indicate the study’s design with a commonly used term in the title or the abstract |
|  |  | (*b*) Provide in the abstract an informative and balanced summary of what was done and what was found |
| Introduction | | |
| Background/rationale | 2 **page 5-introduction** | Explain the scientific background and rationale for the investigation being reported- |
| Objectives | 3 **page 5-introduction** | State specific objectives, including any prespecified hypotheses- |
| Methods | | |
| Study design | 4 **page 6-Methods** | Present key elements of study design early in the paper - |
| Setting | 5 **page 6-Methods** | Describe the setting, locations, and relevant dates, including periods of recruitment, exposure, follow-up, and data collection |
| Participants | 6 **page 6-Methods** | (*a*) Give the eligibility criteria, and the sources and methods of selection of participants. Describe methods of follow-up |
|  |  | (*b*) For matched studies, give matching criteria and number of exposed and unexposed- |
| Variables | 7 **page 6-7-Methods** | Clearly define all outcomes, exposures, predictors, potential confounders, and effect modifiers. Give diagnostic criteria, if applicable |
| Data sources/ measurement | 8* **page 6-7-Methods**, Additional File 2: Fig. S1 | For each variable of interest, give sources of data and details of methods of assessment (measurement). Describe comparability of assessment methods if there is more than one group |
| Bias | 9 **pages 6-7- Methods** | Describe any efforts to address potential sources of bias- |
| Study size | 10 **page 6-Methods** | Explain how the study size was arrived at- |
| Quantitative variables | 11 **page 7-Methods** | Explain how quantitative variables were handled in the analyses. If applicable, describe which groupings were chosen and why- |
| Statistical methods | 12  **page 8-Methods**, Additional File 2: Table S2 | (*a*) Describe all statistical methods, including those used to control for confounding |
|  |  | (*b*) Describe any methods used to examine subgroups and interactions |
|  |  | (*c*) Explain how missing data were addressed |
|  |  | (*d*) If applicable, explain how loss to follow-up was addressed |
|  |  | (*e*) Describe any sensitivity analyses |
| Results | | |
| Participants | 13*  **page 9-Results**  Table 1, Additional File 2: Table S1, and Figure 1 | (a) Report numbers of individuals at each stage of study—eg numbers potentially eligible, examined for eligibility, confirmed eligible, included in the study, completing follow-up, and analysed |
|  |  | (b) Give reasons for non-participation at each stage |
|  |  | (c) Consider use of a flow diagram |
| Descriptive data | 14*  **page 9-Results,**  Table 1 | (a) Give characteristics of study participants (eg demographic, clinical, social) and information on exposures and potential confounders |
|  |  | (b) Indicate number of participants with missing data for each variable of interest |
|  |  | (c) Summarise follow-up time (eg, average and total amount) |
| Outcome data | 15*  **page 9-11-Results**  Table 2, Figures 2, 3, Additional File 2: Fig. S2 | Report numbers of outcome events or summary measures over time |
| Main results | 16  **page 10-11-Results**  Table 3 | (*a*) Give unadjusted estimates and, if applicable, confounder-adjusted estimates and their precision (eg, 95% confidence interval). Make clear which confounders were adjusted for and why they were included |
|  |  | (*b*) Report category boundaries when continuous variables were categorized |
|  |  | (*c*) If relevant, consider translating estimates of relative risk into absolute risk for a meaningful time period |
| Other analyses | 17  **page 10-Results** | Report other analyses done—eg analyses of subgroups and interactions, and sensitivity analyses |
| Discussion | | |
| Key results | 18  **page 11** | Summarise key results with reference to study objectives |
| Limitations | 19  **page 12-13-Discussion** | Discuss limitations of the study, taking into account sources of potential bias or imprecision. Discuss both direction and magnitude of any potential bias |
| Interpretation | 20  **page 11-13-Discussion** | Give a cautious overall interpretation of results considering objectives, limitations, multiplicity of analyses, results from similar studies, and other relevant evidence |
| Generalisability | 21  **page 13-Discussion** | Discuss the generalisability (external validity) of the study results |
| Other information | | |
| Funding | 22  **page 2** | Give the source of funding and the role of the funders for the present study and, if applicable, for the original study on which the present article is based |

*Give information separately for exposed and unexposed groups.
