# Supplementary material for: Long COVID 12 months after discharge: persistent symptoms in patients hospitalised due to COVID-19 and patients hospitalised due to other causes—a multicentre cohort study
Source: BMC Med. 2022 Feb 23;20:92. doi: 10.1186/s12916-022-02292-6 (PMC8863509; doi:10.1186/s12916-022-02292-6)

**Additional File 2.**

Fig. S1. Consideration of each reported sequelae or persistent symptoms (SPS) collected by telephone to distinguish between previous, prevalent, and incident SPS.


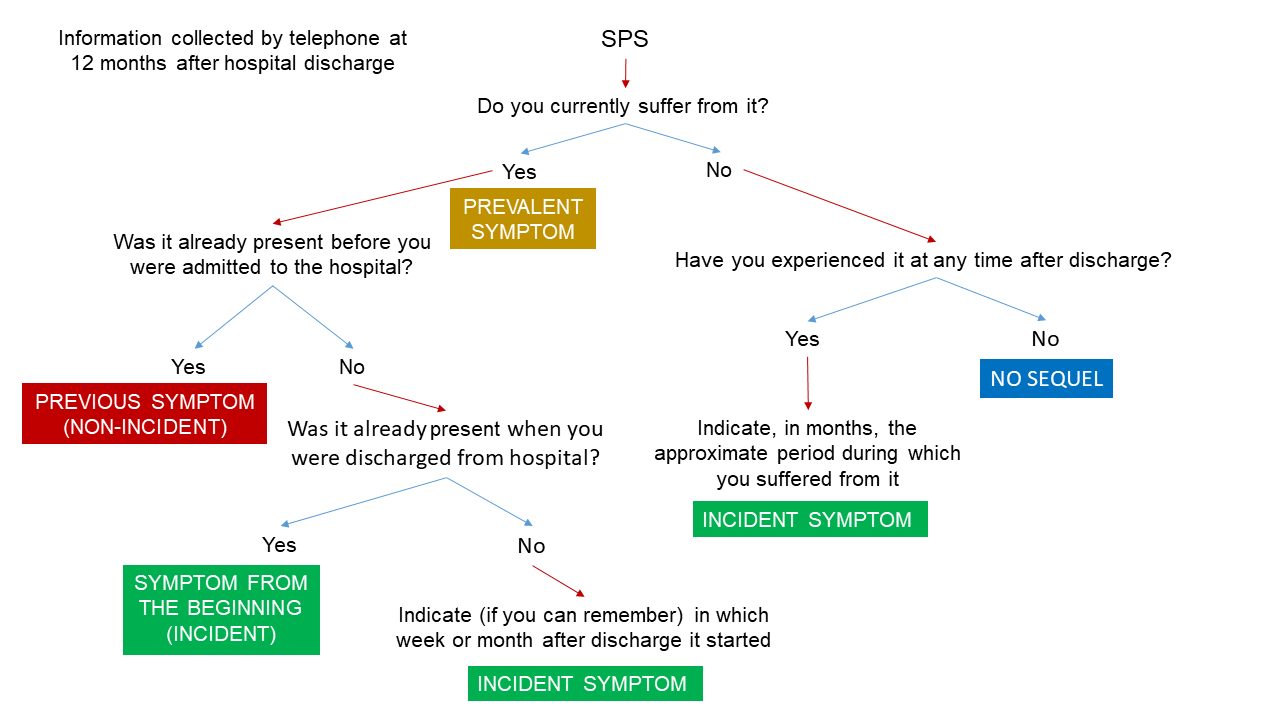


This scheme was followed for each SPS in each patient. As noted in the figure, an SPS could be prevalent (at 12 months after discharge) and incident, only prevalent but not incident (if presented before hospitalization), only incident but not prevalent, or neither incident nor prevalent (no sequel). We asked for all potential SPS considered in the Spanish modified version of the open-access Case Report Form of the Clinical Characterization Protocol for Severe Emerging Infections of the International Severe Acute Respiratory and Emerging Infection Consortium (ISARIC).

Table S1. Commands used for the diagrams with R software.

| **Diagram** | **Commands** |
| --- | --- |
| Sankey diagram (Figure 2) | library(tidyverse)  library(ggplot2)  library(rio)  library(ggalluvial)  db1<-import("C:/Users/GL553V/Documents/R/Secuelas/db/secuelas.xlsx")  db1$sex <- as.factor(db1$sex)  db1$ID <- db1$ID%>% as.factor()  #Select patients hospitalized due to COVID-19 that showed SPS at 12 months after discharge.  db2<-db1 %>% filter(sequel_12m=="1")  #Select those having at least one of the five most frequent types of SPS  db2 <-db2 %>% filter(pneumo=="1" \|  general=="1"\|  neuro=="1" \|  mentalhealth=="1" \|  cardio =="1")  db2<-db2 %>%  select(ID, sex, pneumo, general, neuro, mentalhealth, cardio)  db3<-db2 %>% select(ID, pneumo, general, neuro, mentalhealth, cardio)  db3<-as_tibble(db3)  db3<-db3 %>% group_by(ID)  #One observation per SPS  db3_longer <-db3 %>%  pivot_longer(!ID, names_to = "typeofsps", values_to = "count")  db4<-db3_longer %>% filter(count=="1")  db5<-left_join(db4, db2,  by="ID”)  db6<-db5 %>% select(ID, sex, typeofsps, count)  db6$typeofsps <- db6$typeofsps %>% as.factor()  #Translate labels to complete English terms  levels(db6$typeofsps)<- list(  Cardiovascular="cardio",  "Mental health" = "mentalhealth",  Neurological = "neuro",  Respiratory = "pneumo",  Systemic = "general”)  levels(db6$sex)<- list(  Women = "2",  Men = "1")  #Plot the Sankey diagram  ggplot(as.data.frame(db6),  aes(y = count,  axis1 = typeofsps, axis2 = sex)) +  geom_alluvium(aes(fill = typeofsps),  width = 1/12,  reverse = FALSE) +  theme_minimal()+  guides(fill = "none") +  geom_stratum(width = 1/6, fill="white", col="grey80", size=1,  reverse = FALSE) +  geom_text(stat = "stratum",  aes(label = after_stat(stratum)),  reverse = FALSE,  size=5.2) +  scale_x_continuous(breaks = 1:2, labels = c("Type of SPS",  "Sex")) +  theme(  axis.text.x = element_text(size=18, colour="grey20"),  axis.text.y = element_blank(),  axis.title.y = element_blank(),  axis.title=element_text(size=18),  panel.grid = element_blank() ) |
| Heat map  (Figure 3) | library(tidyverse)  library(ggplot2)  library(rio)  db1<-import("C:/Users/GL553V/Documents/R/Secuelas/db/secuelas.xlsx")  db1$age <- as.numeric(db1$edad)  #Select patients hospitalized due to COVID-19 that showed SPS at 12 months  db2<-db1 %>% filter(secuela_12m=="1")  db3<-db2 %>% select(pneumo, general, neuro, mentalhealth, haemato, derma, nephro, uro, endocr, orl, ophthal, digest, cardio, superinf, age)  #Assign patients hospitalized due to COVID-19 that showed SPS at 12 months to age groups  db3$age_group<- cut(db3$age, c(18, 39, 49, 59, 69, 79, 100) )  levels(db3$age_group)<-list(  "18-39" = "(18,39]",  "40-49"= "(39,49]",  "50-59"= "(49,59]",  "60-69"= "(59,69]",  "70-79"= "(69,79]",  "80+"= "(79,100]")  db3<-as_tibble(db3)  db3<-db3 %>% select(-age)  #Calculate the number of SPS per type of SPS and age group  db3 <- db3 %>% group_by(age_group) %>%  summarise(pneumo=sum(pneumo),  general=sum(general),  neuro=sum(neuro),  mentalhealth=sum(mentalhealth),  haemato=sum(haemato),  derma=sum(derma),  nephro=sum(nephro),  uro=sum(uro),  endrocr=sum(endocr),  orl=sum(orl),  ophthal=sum(ophthal),  digest=sum(digest),  cardio=sum(cardio),  superinf=sum(superinf) )  db3_longer <-db3 %>%  pivot_longer(!age_group, names_to = "sps", values_to = "count")  db3_longer<-as.data.frame(db3_longer)  db3_longer$sps <-as.factor(db3_longer$sps)  db4 <-db3_longer %>% group_by(age_group) %>%  mutate(sumsps = sum(count) )  #Translate labels to complete English terms  levels(db4$sps) <- list(  Urological = "uro",  Systemic = "general",  Respiratory="pneumo",  Otorhinolaryngological = "orl",  Ophtalmological = "ophthal",  Neurological="neurol",  Nephrological = "nephro",  "Mental health"="mentalhealth",  Infection="superinf",  Haematological="haemato",  Digestive = "digest",  Dermatological = "derma",  Cardiovascular = "cardio")  #Plot the heat map  ggplot(db4, aes(x = age_group, y = sps, fill = (count/sumsps)*100) ) +  geom_tile(color="transparent", size=1)+  theme_minimal()+  geom_text (aes(label=round( (count*100/sumsps),1) ), size=4)+  labs(x="Age group (years)", y="Sequelae", fill="Proportion of patients with sequelae (%)" ) +  theme(axis.text=element_text(size=13),  axis.title=element_text(size=14,face="bold"),  legend.title=element_text(size = 13),  legend.text=element_text(size = 11),  legend.position = "top",  panel.grid = element_blank() ) +  scale_fill_gradient(low = "white", high = "purple") |

Table S2. Causes of hospital admission in the non-exposed cohort (n = 453).

| **Service of admission** | **Cause of admission** | **n** |
| --- | --- | --- |
| Angiology and vascular surgery | Total | **14 (3.1)** |
|  | Deep vein thrombosis | 5 |
|  | Arterial insufficiency | 3 |
|  | Abdominal aortic aneurysm | 3 |
|  | Chronic ulcer | 2 |
|  | Diabetic foot | 1 |
| Cardiology | Total, n (%) | **29 (6.4)** |
|  | Acute myocardial infarction | 8 |
|  | Heart failure | 6 |
|  | Valvulopathy | 4 |
|  | Pulmonary embolism with *cor pulmonale* | 3 |
|  | Angina pectoris | 3 |
|  | Atrioventricular block | 2 |
|  | Atrial fibrillation | 2 |
|  | Supraventricular tachycardia | 1 |
| Critical care | Total | **8 (1.8)** |
|  | Acute myocardial infarction | 4 |
|  | Hypertensive crisis | 2 |
|  | Endocarditis | 1 |
|  | Haemorrhagic gastric ulcer | 1 |
| Dermatology | Total | **8 (1.8)** |
|  | Melanoma | 3 |
|  | Basal cell carcinoma | 3 |
|  | Squamous cell carcinoma | 2 |
| Digestive (Gastroenterology) | Total | **22 (4.9)** |
|  | Duodenal ulcer | 6 |
|  | Crohn’s disease | 3 |
|  | Gastroenteritis | 3 |
|  | Diverticulitis | 3 |
|  | Diverticulosis | 3 |
|  | Hepatic insufficiency | 2 |
|  | Portal hypertension | 1 |
|  | Gastrointestinal haemorrhage | 1 |
| Endocrinology | Total | **4 (0.9)** |
|  | Complications of diabetes mellitus type 1 | 2 |
|  | Complications of thyroid diseases | 2 |
| General surgery | Total, n (%) | **58 (12.8)** |
|  | Biliary calculus | 10 |
|  | Breast cancer | 7 |
|  | Colorectal cancer | 6 |
|  | Appendicitis | 6 |
|  | Acute pancreatitis | 5 |
|  | Complications of diabetes mellitus | 5 |
|  | Post-surgical infection | 4 |
|  | Inguinal hernia | 4 |
|  | Anal abscess | 3 |
|  | Liver cancer | 2 |
|  | Biliary tract cancer | 1 |
|  | Thyroid cancer | 1 |
|  | Intestinal obstruction | 1 |
|  | Retroperitoneal carcinoma | 1 |
|  | Sepsis | 1 |
|  | Post-surgical haemorrhage | 1 |
| Infectious diseases | Total | **19 (4.2)** |
|  | Sepsis | 3 |
|  | Fever of unknown origin | 3 |
|  | Non-confirmed suspicion of COVID-19 | 3 |
|  | Zoonosis | 3 |
|  | Other viral respiratory infections | 3 |
|  | Tuberculosis | 1 |
|  | Meningitis | 1 |
|  | Human Immunodeficiency Virus | 1 |
|  | Device-associated infection | 1 |
| Internal medicine | Total | **18 (4.0)** |
|  | Chronic pluripathology | 10 |
|  | Substance abuse toxic effects | 4 |
|  | Inhalational pneumonitis | 2 |
|  | Secondary effects of treatments | 2 |
| Gynaecology | Total | **8 (1.8)** |
|  | Gynaecologic infection | 4 |
|  | Endometrial cancer | 1 |
|  | Ovarian cancer | 1 |
|  | Endometriosis | 1 |
|  | Ovarian cyst | 1 |
| Haematology | Total | **6 (1.3)** |
|  | Lymphoma | 2 |
|  | Leukaemia | 2 |
|  | Chemotherapy-associated agranulocytosis | 2 |
| Nephrology | Total, n (%) | **10 (2.2)** |
|  | Chronic renal disease | 4 |
|  | Acute renal failure | 2 |
|  | Drug-induced nephropathy | 2 |
|  | Glomerulonephritis | 1 |
|  | Kidney cancer | 1 |
| Neurology | Total | **16 (7.1)** |
|  | Stroke | 10 |
|  | Epilepsy | 5 |
|  | Multiple sclerosis | 1 |
| Neurosurgery | Total | **6 (1.3)** |
|  | Intracerebral haemorrhage | 2 |
|  | Brain cancer | 2 |
|  | Cerebral aneurysm | 2 |
| Obstetrics | Total | **92 (20.3)** |
|  | Complications during childbirth | 15 |
|  | Post-term pregnancy | 14 |
|  | Anomaly of labour dynamics | 13 |
|  | Prolonged labour | 9 |
|  | Foetal heart rhythm abnormality | 8 |
|  | Pregnancy complications | 8 |
|  | Retained abortion | 7 |
|  | Perineal tear | 6 |
|  | Diseases that complicate childbirth | 5 |
|  | Preterm birth | 3 |
|  | Preeclampsia | 3 |
|  | Thrombophilia during pregnancy | 1 |
| Oncology | Total | **10 (2.2)** |
|  | Antineoplastic chemotherapy | 8 |
|  | Cancer-related complications | 2 |
| Ophthalmology | Total | **13 (2.9)** |
|  | Retinal detachment | 6 |
|  | Strabismus | 4 |
|  | Corneal ulcer | 2 |
|  | Glaucoma | 1 |
| Otorhinolaryngology | Total | **7 (1.5)** |
|  | Peritonsillar abscess | 4 |
|  | Retropharyngeal abscess | 2 |
|  | Localised adenomegaly | 1 |
| Pneumology | Total | **32** |
|  | Bacterial pneumonia | 6 |
|  | Chronic obstructive pulmonary disease exacerbation | 6 |
|  | Influenza infection | 5 |
|  | Interstitial lung disease | 4 |
|  | Lung cancer | 4 |
|  | Asthma exacerbation | 3 |
|  | Larynx cancer | 1 |
|  | Acute bronchiolitis | 1 |
|  | Emphysema | 1 |
|  | Inhalational pneumonitis | 1 |
| Psychiatry | Total | **7 (1.5)** |
|  | Major depressive disorder | 3 |
|  | Substance dependence | 3 |
|  | Acute intoxication | 1 |
| Traumatology | Total | **36 (7.9)** |
|  | Hip fracture | 10 |
|  | Arthrosis complications | 6 |
|  | Radius fracture | 6 |
|  | Intervertebral disc herniation | 4 |
|  | Tibia fracture | 3 |
|  | Skull fracture | 2 |
|  | Costal fracture | 2 |
|  | Humerus fracture | 1 |
|  | Peroneal fracture | 1 |
|  | Osteomyelitis | 1 |
| Urology | Total, n (%) | **30 (6.6)** |
|  | Urinary tract calculus | 10 |
|  | Urinary tract infection | 7 |
|  | Prostate cancer | 4 |
|  | Benign prostatic hyperplasia | 4 |
|  | Bladder cancer | 2 |
|  | Pyelonephritis | 2 |
|  | Hydronephrosis | 1 |

Fig. S2. Correlation matrix of the association between the most frequent sequelae and persistent symptoms (SPS).


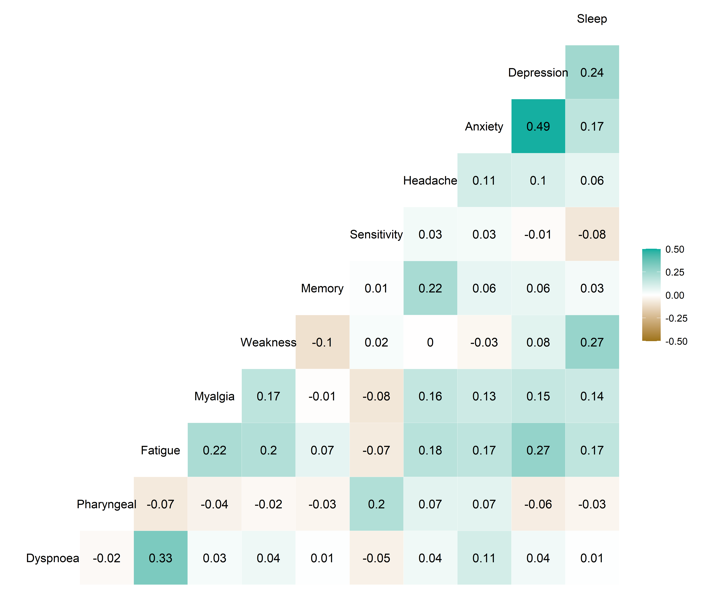

Supplement: Supplementary file 2 — Additional file 2: Table S1. Causes of admission of the non-exposed cohort (n = 453). Table S2. Commands used for the diagrams with R software. Fig. S1. Consideration of each reported sequelae or persistent symptoms (SPS) collected by telephone to distinguish between previous, prevalent, and incident SPS. Fig. S2. Correlation matrix of the association between the most frequent sequelae and persistent symptoms (SPS). [file 12916_2022_2292_MOESM2_ESM.docx]
